# Preparation and Effect of CO_2_ Response Gel for Plugging Low-Permeability Reservoirs

**DOI:** 10.3390/gels10070449

**Published:** 2024-07-09

**Authors:** Huipeng Wang, Changhua Yang, Yongwei Zhang, Chen Wang

**Affiliations:** School of Petroleum Engineering, Xi’an Shiyou University, Xi’an 710065, China; 15965803805@163.com (H.W.); 13571453944@163.com (Y.Z.); 15238733188@163.com (C.W.)

**Keywords:** gas channeling, CO_2_ response gel, response surface, three-dimensional network structure

## Abstract

In order to solve the problem of gas channeling during CO_2_ flooding in low-permeability reservoirs, a novel CO_2_ responsive gel channeling system was prepared by using carrageenan, branched polyethylene imide and ethylenediamine under laboratory conditions. Based on the Box–Behnken response surface design method, the optimal synthesis concentration of the system was 0.5 wt% carrageenan, 2.5 wt% branchized polyethylenimide and 6.5 wt% ethylenediamine. The micromorphology of the system before and after response was characterized by scanning electron microscopy. The rheology and dehydration rate were tested under different conditions. The channeling performance and enhanced oil recovery effect of the gel system were simulated by a core displacement experiment. The experimental results show that the internal structure of the system changes from a disordered, smooth and loosely separated lamellae structure to a more uniform, complete and orderly three-dimensional network structure after exposure to CO_2_. The viscosity of the system was similar to aqueous solution before contact with CO_2_ and showed viscoelastic solid properties after contact with CO_2_. The experiment employing dehydration rates at different temperatures showed that the internal structure of the gel would change at a high temperature, but the gel system had a certain self-healing ability. The results of the displacement experiment show that the plugging rate of the gel system is stable at 85.32% after CO_2_ contact, and the recovery rate is increased by 17.06%, which provides an important guide for the development of low-permeability reservoirs.

## 1. Introduction

Of the known reservoir reserves, low-permeability reservoirs account for about 38% of the world’s total, and China accounts for about two-thirds of them [1,2]. The characteristics of small pore throat size, complex pore structure, natural micro-fracture development, and the wide spatial scale distribution of fractures and pore throats in low-permeability reservoirs make them more difficult to exploit [3,4]. The practice at home and abroad shows that CO_2_ flooding is one of the key technologies used for the effective exploitation of low-permeability reservoirs [5]. The high fluidity, viscosity reduction, volume expansion and interfacial tension reduction of CO_2_ gas make it more effective than water flooding in the process of oil flooding to reduce the residual oil saturation in the pores [6,7]. However, the CO_2_ flooding method has some disadvantages, such as low sweep efficiency, mainly due to the heterogeneity of the reservoir, and it is easy for gas to form a channel in the hyperpermeability area, and the viscous fingering and gravity overlap are caused by the viscosity difference and density difference between crude oil and CO_2_. The main reason for CO_2_ displacement channeling is that the viscosity of CO_2_ is much lower than that of formation water and crude oil, resulting in the difference in fluidity between them [8]. This difference causes the injected CO_2_ to bypass the crude oil and cross-flow occurs, reducing the sweep efficiency. Secondly, the heterogeneity of the reservoir means the injected CO_2_ tends to form a channeling channel along the high-permeability zone, which further affects the displacement effect. In addition, there are also problems such as gravity overlap, oil and gas viscosity differences, and non-homogeneous layer capillary forces, which not only limit the gas displacement efficiency but also increase the difficulty of plugging gas channeling [9].

To solve the above problems, domestic and foreign scholars have accumulated numerous anti-gas channeling methods and applied them to low-permeability reservoirs at home and abroad on a large scale [10]. At present, the main methods to prevent CO_2_ gas channeling include alternating injections of water and gas (WAG), polymer gel channeling [11,12], chemical precipitation, CO_2_ foam [13] and CO_2_ thickening [14]. These methods have played a positive role in controlling gas channeling, but there are also great limitations. Alternate injections of water and air are not suitable under severe heterogeneous conditions because the fluidity instability during gas injection can lead to cavitation and device corrosion problems [14]. Gel injection is limited by its performance, and it is difficult to effectively plug deep reservoirs. The controllability of chemical precipitation is poor; it is difficult to effectively control the improvement effect, and this may cause damage to the reservoir [15]. The strength of CO_2_ foam is low; it is difficult to control foam formation under reservoir conditions, and it is not suitable for severely heterogeneous conditions. CO_2_ thickening methods are costly and complex to configure. In recent years, a new class of smart polymers, namely CO_2_ stimulus responsive polymers, has emerged [16]. These polymers have a special structure and contain groups that can respond to CO_2_, such as amidinoyl, guanidine and amine groups [17,18]. Li’s team synthesized a CO_2_-responsive long-chain polyamine (ODPTA) and applied this long-chain polyamine to foam generation, achieving excellent plugging performance [19]. Although CO_2_-responsive foams have shown remarkable results in enhancing WAG projects, the resulting foams are often thermodynamically unstable and have a weak plugging strength compared to gels, limiting their application in the field. For the first time, Wan-fen Pu et al. [20] prepared polyacrylamide colloid (PAASP) by using methylene bisacrylamide as a crosslinking agent. Subsequently, they polymerized and cross-linked CO_2_ response monomers, acrylamide, etc., with the first network in situ to form an interpenetrating network polymeric gel (IPN-ASAP), and they revealed its response mechanism. The study showed that the protonation reaction occurred after the contact of IPN-ASAP with CO_2_, which made the polymer coil expand. At the same time, due to the hydrophilic change and electrostatic repulsion, more water molecules entered the gel interior and increased its particle size. In laboratory EOR experiments, the injection pressure of IPN-ASAP increased from 5.0 KPa before exposure to CO_2_ to 342.0 KPa. The injected CO_2_ successfully penetrated the matrix and displaced the oil, resulting in an 8.2% increase in recovery in the tight core (permeability 0.85 × 10^−3^ μm^2^). Aiming at the problem of gas channeling in the late stage of a low-permeability reservoir, Demingley et al. [21] synthesized a CO_2_-responsive gel in the laboratory. The results of environmental scanning electron microscopy showed the formation of three-dimensional network aggregates. After protonation, it was confirmed that bola-type amines bridge two anionic surfactants through non-covalent force to form pseudo-gemini surfactants. This causes wormlike micelles to aggregate and tangle with each other, eventually forming a gel system. The results show that the sealing efficiency of the gel system is more than 90%. Yong-Guang Jia et al. [22] synthesized hydrogels with self-healing properties using cholic acid dimers as guest crosslinkers and a β-cyclodextrin polymer as the main body. To provide the system responsiveness, they introduced protonable benzimidazole. Under alternating CO_2_/N_2_ bubbling, the reversible transition between solution and gel was realized due to the dynamic complexation between cholic acid, benzimidazole and β-cyclodextrin. These laboratory experiments demonstrate the potential of CO_2_-responsive polymers in improving reservoir heterogeneity and improving oil displacement efficiency. However, given the application in oilfield construction sites, the monomer cost of CO_2_-responsive polymers is high, and the synthesis of polymers with complex supramolecular structures is cumbersome and rigorous for industrial production conditions. Therefore, much research is still needed to verify the applicability of CO_2_-responsive polymers in the oilfield field.

As one of the important components of CO_2_ geological utilization in CCUS technology, CO_2_ flooding is also one of the key technologies for enhanced oil recovery, so it is particularly important to solve the gas channeling problem [23]. CO_2_ flooding can effectively utilize and store a large amount of CO_2_, thus reducing emissions to the atmosphere, which is of great significance for mitigating climate change. A novel CO_2_ response gel channeling system was prepared by using carreenan, branched polyethylenimide and ethylenediamine. The protonation process of amine functional groups was understood by means of microscopic morphology characterization; the reason for the sharp increase in the viscosity of the system after contact with CO_2_ was revealed from a microscopic point of view; and the rheological properties of the CO_2_ response gel system and the performance of plugging to enhance oil recovery were clarified [24]. This provides a new way to solve the problem of gas channeling in CO_2_ flooding technology in low-permeability reservoirs and also brings important development opportunities for CO_2_ capture, utilization and storage (CCUS) [25].

## 2. Results and Discussion

### 2.1. Preparation of the Gel System

**Figure 1:**
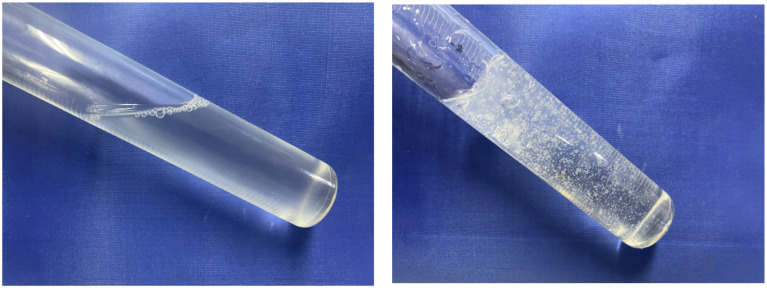
Changes before and after CO_2_ exposure.

**Figure 2:**
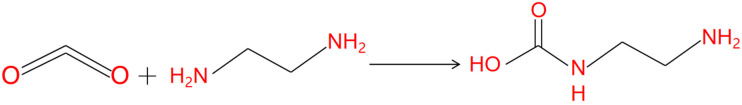
Ethylenediamine principle diagram of the reaction with CO_2_.

The physical drawings before and after the response of the three aqueous systems to CO_2_ exposure are shown in Figure 1. Before contact with CO_2_, we have a transparent clear solution with a low viscosity, close to water; however, once fully in contact with CO_2_, the viscosity of the system rises sharply, forming a CO_2_ responsive gel sealing system. Carrageenan is a negatively charged biopolymer that can interact with positively charged polymers, and carrageenan itself is a thermally reversible physical gel. Branched polyethyleneimine is a polycation, and the polymer chain contains a large number of amine groups, such as primary amines, secondary amines, tertiary amines, etc. [26,27], in the CO_2_ due to the acidic conditions of protonation, thereby interacting with the negatively charged polyanion, resulting in the ordered cross-linking of the polymer chain. At the same time, ethylenediamine will chemically react with CO_2_ to form organic amine salt (ammonium carbamate) with high viscosity, which further improves the strength of the gel system. The reaction principle is shown in Figure 2 [28].

### 2.2. Concentration Optimization of Gel System

#### 2.2.1. Results of Response Surface Experiment Design

According to the results of the single-factor experiment, three single-factor variables with the greatest influence on the gel strength were selected. Based on the Box–Behnken response surface design method, an experiment with three factors, three levels and seventeen experimental points was designed. The design and results of the response surface experiment are shown in Table 1.

#### 2.2.2. Model Establishment and Significance Analysis

Design Expert version 13 software was used to analyze the variance of the experimental results of the response gel, and the quadratic polynomial regression equation obtained by multiple regression fitting of the data in Table 2 is as follows:

Y = 9.4 + 0.1375A − 0.0125B + 0.25C + 0.1AB + 0.025AC + 0.725BC − 1.63A^2^ − 1.23B^2^ − 0.2C^2^. (1)

In Formula (1), Y—Intensity Score, points; A—Carrageenan Addition, wt%; B—Branched Polyvinylimide Addition, wt%; and C—Ethylenediamine Addition, wt%.

The results of the regression model ANOVA are shown in Table 2 [29].

Table 2 shows that significant items for A and C^2^. The most significant terms were C, BC, A^2^ and B^2^. The insignificant terms were B, AB, and AC. The *p*-value of the model was less than 0.0001, indicating that the model was extremely significant. The loss of fit term was not significant (*p* = 0.9559 > 0.05), indicating that the degree of nonlinear fitting was relatively high. The coefficient of determination (R^2^ = 0.9951) indicated that the actual experimental values were well correlated with the predicted values. Therefore, this model can better reflect the relationship between monomer addition and gel strength in the system and predict the best reaction conditions. According to the *F* value, the order of influence of the three factors on the gel strength score is C > A > B; that is, the amount of ethylenediamine added > the amount of carrageenan added > the amount of branched polyethylenimine added.

**Figure 3:**
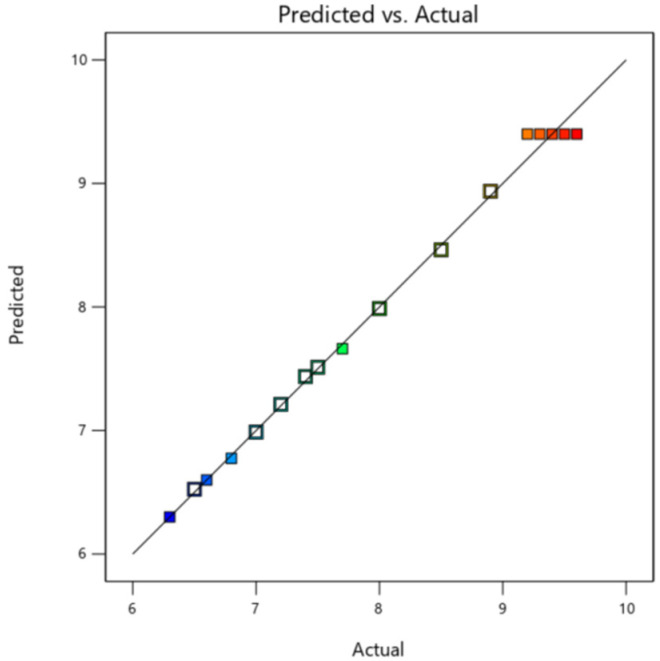
Comparison between the predicted values of the model and the actual values.

Figure 3 shows the comparison between the predicted values of the model and the actual values. Figure 3 shows that the actual and predicted values of the established model are almost on the line Y = X, indicating that this quadratic regression model can be used for response surface analysis and the optimization of CO_2_ responsive gels.

#### 2.2.3. Response Surface Analysis

**Figure 4:**
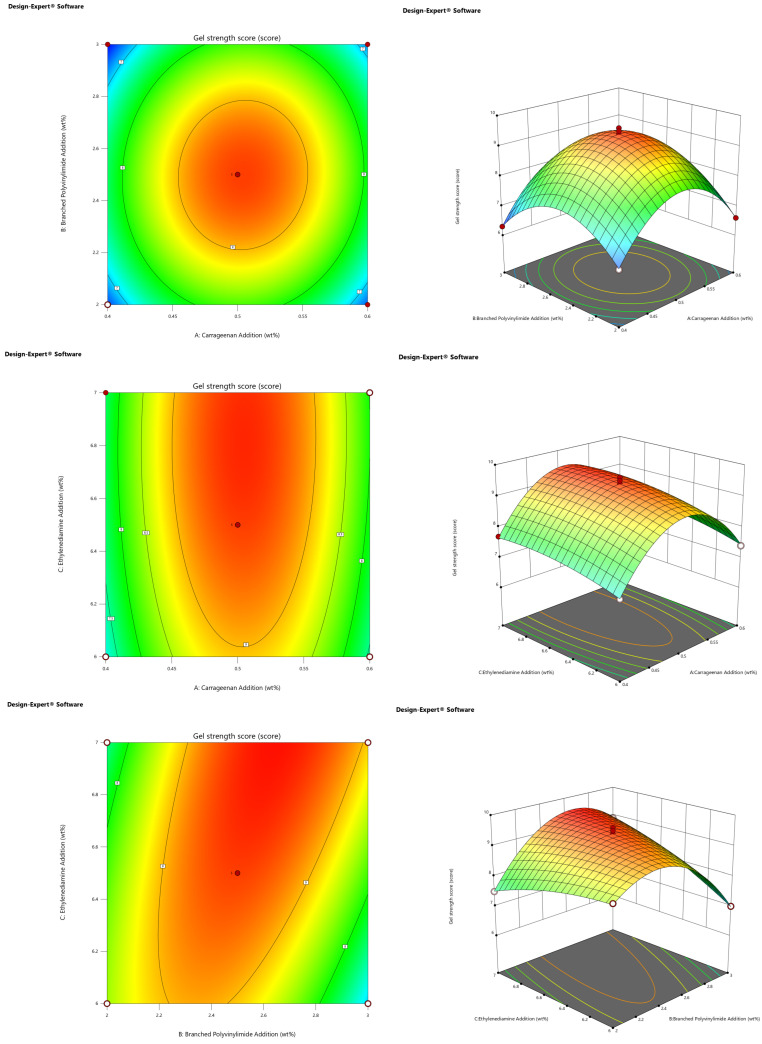
Contour plot and response surface plot of the interaction between the three factors.

The use of the Design Expert software was used to show interactions between various factors, focusing on their impact on the CO_2_ response gel strength of the contour plots and response surface figures, as shown in Figure 4 [30].

Figure 4 shows that response surface figure of the degree of curvature of curve can reflect the study of the strength of the factors that affect the results. The more curved the curve, the greater the influence of this factor on the results. If the contour shows an oval shape, it means that there is a significant interaction between the study factors. If it is circular, the interaction is less significant. Therefore, based on the above analysis of contour lines and response surface plots, it can be concluded that the interaction between the dosage of ramped polyethylenimine and ethylenediamine was extremely significant (*p* < 0.01), and the interaction between other factors was not significant. The single-factor addition of ethylenediamine had the most obvious effect on the strength of the response gel, followed by the addition of carrageenan, and the least effect was produced by the addition of branched polyethylenimine.

#### 2.2.4. Prediction and Verification of Optimal Process Conditions

The experimental data were optimized and predicted by Design Expert software. The best formula of CO_2_ response gel was 0.506 wt% for carrageenan, 2.548 wt% for polyethylenimine, and 6.602 wt% for ethylenediamine, and the predicted value of CO_2_ response gel strength score was 9.559. Combined with the actual operation, the selection of a carrageenan added amount of 0.5 wt%, a branched polyethylene imine added amount of 2.5 wt %, and an ethylenediamine added amount of 6.5 wt % is close to the model prediction and shows that the model can be used for the CO_2_ response to the strength of gel process optimization.

### 2.3. Microstructure of the CO_2_ Responsive Gel

**Figure 5:**
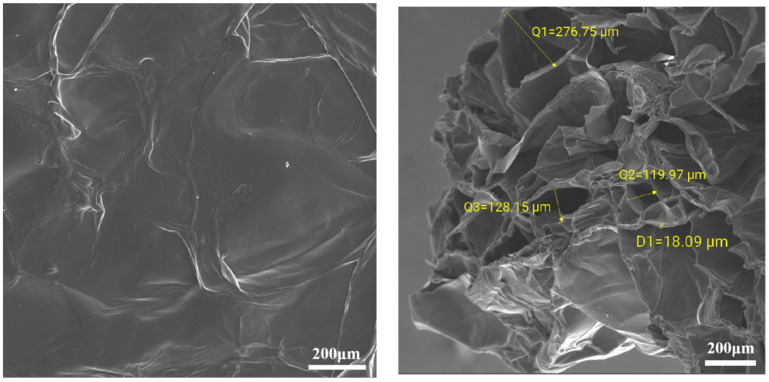
Electron microscopic images of the CO_2_ responsive gel system before and after response.

Using scanning electron microscopy (SEM) with a carrageenan added amount of 0.5 wt%, a branched polyethylene imine added amount of 2.5 wt%, and an ethylenediamine added amount of 6.5 wt%, as well as a volume ratio of 2:1:1 for gel sealing channeling system components before and after contact with CO_2_, we can observe the response of the internal structure through electron microscopy (SEM) images, as shown in Figure 5.

As shown in Figure 5, in the electron microscope picture of the system before CO_2_ response, it can be seen that the lamellar structures are disorderly and loosely separated from each other, and the surface is relatively flat and smooth [31]. Before CO_2_ gas, carrageenan and branched polyethylenimine, two long-chain polymers, are physically wrapped, the connection points are not evenly distributed, and CO_2_ cannot chemically react with ethylene diamine. Due to the low degree of cross-linking, the internal support is insufficient, and the structure is easily damaged by the influence of the external environment. With the penetration of CO_2_ gas, the polycation undergoes protonation and interacts with the negative group on the polyanion. This electrostatic interaction causes the whole system to spread out evenly, forming a more uniform, complete and neatly arranged three-dimensional network structure, and the parts are closely connected to each other. In addition, the CO_2_ and ethylene diamine reaction increases the system viscosity. Therefore, the overall structure of the gel system is more stable. By measuring the distance between the two reticular structures, it was found that the distance between each two reticular structures was between 15 and 130 μm [32], indicating that the reticular structures were closely arranged and the strength of the gel was stronger, and it was not easy to break.

### 2.4. Rheological Test

**Figure 6:**
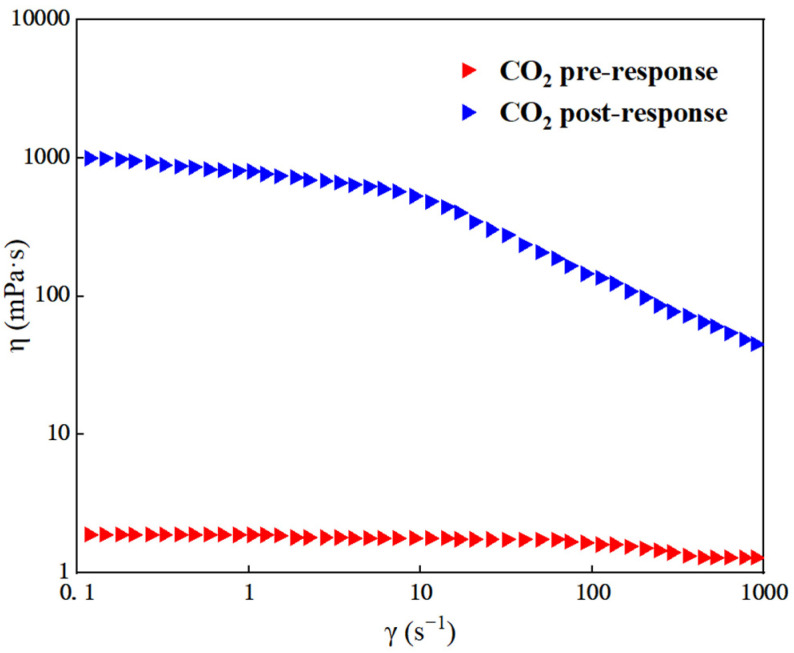
Flow curves of the system before and after CO_2_ response.

**Figure 7:**
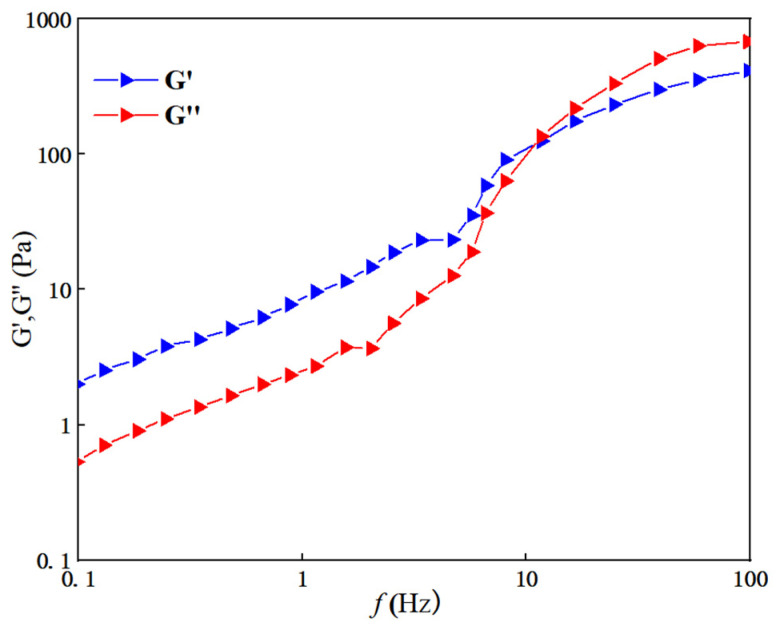
Response gel viscoelastic curves.

Under 30 °C, a carrageenan added amount of 0.5 wt%, a branched polyethylene imine added amount of 2.5 wt%, and an ethylenediamine added amount of 6.5 wt%, as well as a volume ratio of 2:1:1 for system components before and after contact with CO_2_, we can observe the response of viscosity change with the shear rate, as shown in Figure 6. The response gel was scanned at different frequency ranges under a shear stress of 0.2 Pa [33], and the viscoelastic curves are shown in Figure 7.

Figure 6 shows that before the gel system is exposed to CO_2_, the viscosity of the gel is similar to that of water (about 1.7 mPa·s), and the viscosity is almost not affected by the shear rate, which belongs to a typical Newtonian fluid. However, once in contact with CO_2_, the viscosity of the gel began to decrease with increasing shear rate, showing the characteristic of shear thinning. When the shear rate is low, within 10 s^−1^, the change in viscosity is relatively small and the reaction of the gel is weak. But the gel high-speed shear viscosity will be significantly reduced, eventually becoming steady at about 45 mPa·s.

Figure 7 shows that both the storage modulus G′ and the loss modulus G″ of the CO_2_ response gel increase with the increase in the scanning frequency. In the low-frequency region, the CO_2_ response gel energy storage modulus G′ is greater than the loss modulus G″, which indicates that system gives priority to a viscoelastic solid and has obvious elasticity, and shear oscillation will not destroy the response to the internal structure of the gel. In the high-frequency region, the two curves intersect, which indicates that a local collapse of the internal structure of the response gel begins, gradually changing from a viscoelastic solid to a viscoelastic liquid. At the intersection point, after CO_2_ response gel energy storage modulus G′ is less than the loss modulus G″, the system gives priority to a viscoelastic fluid and the observed system becomes obviously thinner; at the same time, it also has a certain viscosity.

### 2.5. CO_2_ Responds to the Dehydration Rate of the Gel

**Figure 8:**
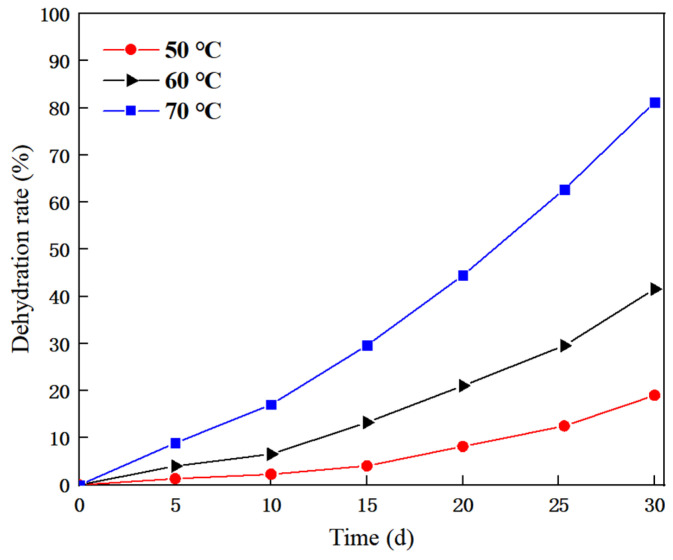
Gel dehydration rates at different temperatures.

The prepared CO_2_ response gel sealing channeling system was placed in an oven at 50 °C, 60 °C, and 70 °C, and the water loss mass of the system was recorded every five days during the aging process, with a total aging time of 30 days. The experimental results are shown in Figure 8.

Figure 8 shows that with the increase in aging days, the dehydration rate at all three temperatures becomes larger. At 50 °C, the dehydration rate remained below 10% for the initial 15 days, while after 30 days, the dehydration rate of the system eventually stabilized at about 18%. At 60 °C, the dehydration rate of the system did not change much during the first 10 days but gradually increased thereafter, eventually reaching around 40% after 30 days. However, at 70 °C, the dehydration rate of the system began to increase significantly on day 5, showing poor water retention, and the final dehydration rate was around 80% after 30 days. This suggests that high temperature may result in changes in the molecular structure of the polymer, such as the cross-linking of the polymer chain or fracture, thus affecting the material properties. It is worth noting that when the temperature returned to room temperature, the gel system returned to the previous state, indicating that the response gel had some self-repair ability [34,35].

### 2.6. Response Gel Gas Channeling Plugging Ability

**Figure 9:**
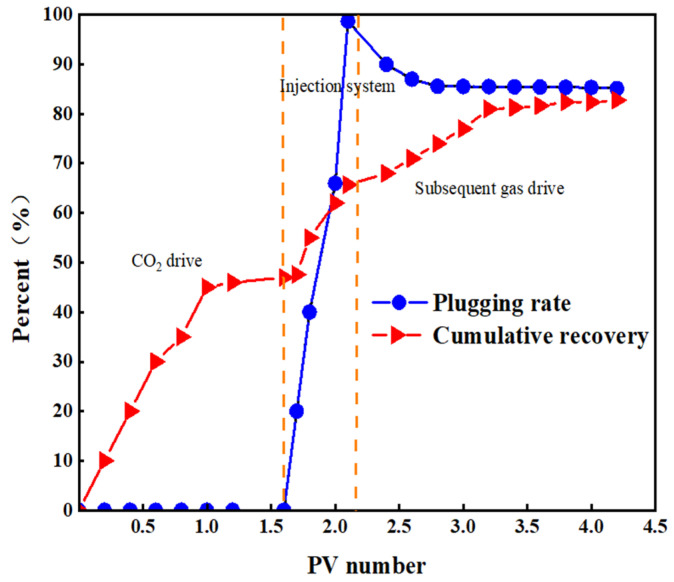
Cumulative recovery and laboratory sealing rate.

**Figure 10:**
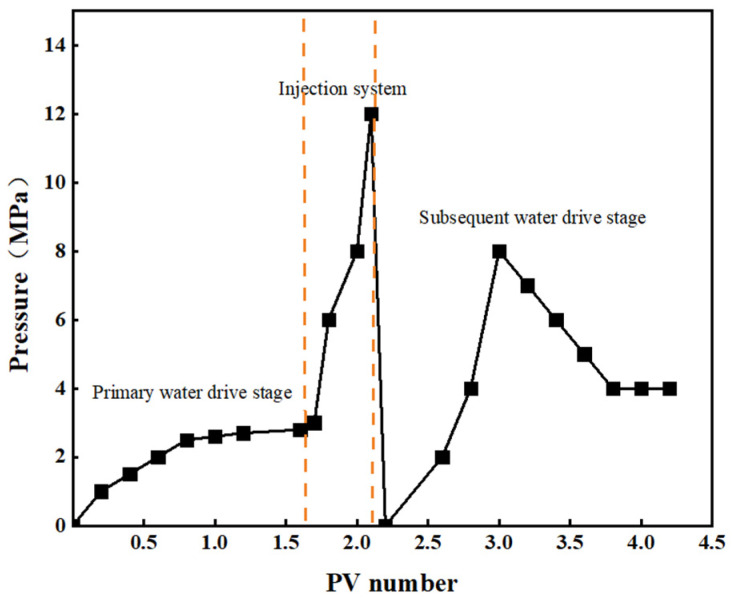
Core displacement pressure changes.

The injectivity and pluggability of the CO_2_-responsive gel were evaluated using a core displacement device, and the experimental results are shown in Figure 9 and Figure 10 [36].

Figure 9 and Figure 10 show that during the primary CO_2_ displacement stage, gas injection was 1.7 times the core pore volume, and subsequent recovery was stable at 47.6%. When a gel system of 0.4 times the pore volume was injected into the system, the pressure at the inlet of the core gripper rose rapidly and finally stabilized at about 12 MPa [37]. This indicates that during the injection process, the gel-based liquid reacts with the CO_2_ gas in the core pores to form a gel system, which effectively seals the large pores in the core, resulting in a recovery rate of 18.13% during the injection phase [38]. Subsequent gas drives the remaining oil in the pore out, and the subsequent stage of gas drives the recovery efficiency up to 17.06%, and then it remained stable. The overall recovery efficiency reached 82.76%. After the CO_2_ contact system, the blocking rate of the laboratory reached 98.73% and then decreased during the gas flooding process, and the final blocking rate stabilized at 85.32%, which indicated that the response gel system could effectively block the gas channelization channel.

## 3. Conclusions

(1) A new CO_2_ response gel sealing and channelizing system was prepared by using carrageenan, polyethylenimine and ethylenediamine. The Box–Behnken response surface design method was used to establish the regression equation of the relationship between the gel strength score and the addition of the three agents, and the optimal formula of the gel system was obtained: carrageenan added 0.5 wt%, branched polyethylenimine added 2.5 wt%, and ethylenediamine added 6.5 wt%.
(2) Scanning electron microscopy was used to characterize the internal structure of the gel sealing and channeling system before and after contact with CO_2_. The surface of the system was smooth before contact with CO_2_. As the CO_2_ gas bubbled into the more uniform, complete and neat rows of the 3D mesh structure, each part was closely connected.
(3) Rheological testing was carried out through the gel system. Before CO_2_ contact, the gel system behaves as a Newtonian fluid with a viscosity of about 1.7 mPa·s, which is almost unaffected by the shear rate. In the low-frequency region, the gel is mainly a viscoelastic solid, and the storage modulus G′ is greater than the loss modulus G″, showing obvious elasticity. In the high-frequency region, with the increase in scanning frequency, the internal structure of the gel changes from a viscoelastic solid to a viscoelastic liquid. The storage modulus G′ is smaller than the loss modulus G″, and the system shows viscous characteristics.
(4) The dehydration rate of the response gel at different temperatures was tested. With the increase in aging time, the dehydration rate of the gel system at different temperatures gradually increased, indicating that a high temperature may cause structural changes in the polymer. However, when the polymer was restored to normal temperature, it returned to the original state, and the gel system showed strong self-repair ability.
(5) In the CO_2_ displacement process, the gel system successfully blocked the macropores in the core, resulting in a recovery rate of 18.13%. A later stage of gas drives the effective expulsion of the remaining oil in the small pore, producing a recovery efficiency up to 17.06%, and the overall recovery rate reached 82.76%. Laboratory results showed that the plugging rate of the response gel system was as high as 98.73% after CO_2_ contact, decreased slightly during gas flooding, and finally stabilized at 85.32%.

## 4. Materials and Methods

### 4.1. Experimental Instruments and Raw Materials

A HAAKE RS600 rheometer was provided by Thermo Fisher Technology (Shanghai, China) Co., LTD., with a magnetic stirring device from Shanghai Jiecheng Experimental Instrument (Shanghai, China) Co., LTD., a blast drying oven from Gongyi Yuhua Instrument (Zhengzhou, China) Co., LTD., and a freeze dryer from Sihuan Scientific Instrument (Tianjin, China) Technology Co., LTD, as well as an electronic analytical balance from Nanjing Donglong Machinery Technology (Nanjing, China) Co., LTD and a field emission scanning electron microscope from FEI Company (Houston, TX, USA).

Ethylenediamine and carrageenan (purity 99.99%, analytical purity) were supplied by Aladdin Reagent Shanghai (Shanghai, China) Co., LTD. Branched polyethylenimine (molecular weight 700,000, 50 wt% aqueous solution) was from Beijing Huawei Ruike Chemical (Beijing, China) Co., LTD. and high-purity CO_2_ gas (purity 99.99%) was provided by Shaanxi Kuaite Gas (Xi’an, China) Co., LTD. Deionized water was homemade in the laboratory [39].

### 4.2. Preparation of CO_2_-Responsive Gel System

A certain concentration of carrageenan solution was prepared by taking a certain amount of carrageenan powder and dissolving it in a certain amount of deionized water under the condition of 50 °C, using a digital-display constant-temperature mixer for about 30 min. Then, a certain amount of branched polyethylenimide aqueous solution was added to an appropriate amount of deionized water and diluted to a specific concentration. The mixture was placed in a digital-display constant-temperature stirring device for about 45 min to prepare a certain concentration of branched polyethylenimide aqueous solution [40]. Finally, a certain amount of ethylenediamine solution was added to an appropriate amount of deionized water and diluted to a specific concentration. The mixture was stirred in a digital-display constant-temperature stirring device for about 20 min to prepare a certain concentration of ethylenediamine aqueous solution. The ratio of the three aqueous solutions was V (Carrageenan solution):V (branched polythylenimine aqueous solution):V (ethylenediamine aqueous solution) = 2:1:1. The solution system was fully stirred in a digital-display constant-temperature stirring device for about 30 min, and the prepared solution system was transferred to a high-temperature test tube. Then, CO_2_ gas was injected at a flow rate of 5 mL/min for 3 min to form a CO_2_ responsive gel system.

### 4.3. System Optimization

The gel system was optimized based on the Box–Behnken response surface design method [41,42]. According to a large number of single-factor experimental results, the optimal concentration of Carrageenan solution was 0.4–0.6 wt% under the condition that three aqueous solutions V (carrageenan solution), V (branched-polyethyleneimine aqueous solution) and V (ethylenediamine aqueous solution) = 2:1:1. The optimal concentration of branched polyethylenimide solution was 2.0–3.0 wt%. The optimal concentration of ethylenediamine aqueous solution is 6.0–7.0 wt%. Therefore, a three-factor, three-level experiment was designed to observe the gel strength based on the GSC visual method proposed by Sydansk, and the gel strength grade was divided into A–J. For convenience, the gel strength score of 1–10 was taken as the response value, and the higher the score, the greater the gel strength. The dosage of carrageenan, branched polyethylenimide and ethylenediamine were selected as variable factors. Test factors and levels are shown in Table 3.

### 4.4. Testing and Characterization

(1) The scanning electron microscope. The experimental pre-and post-response system samples were rapidly quenched in liquid nitrogen to maintain their morphology. The samples were subsequently transferred to a freeze-drying oven and freeze-dried at −75 °C for approximately 36 h to obtain freeze-dried samples of the different systems. Next, an appropriate amount of dry sample was prepared as thin slices, and the samples were sprayed with gold. Finally, conductive glue was used to paste the sample on the sample stage for further experimental analysis.

(2) Rheological test. At 30 °C, a HAAKE RS600 rheometer was used to test the system before and after CO_2_ response at different shear rates ranging from 0.01 to 1000 s^−1^, and the viscosity change was observed. We observed the response to CO_2_ gels in different frequency ranges, measuring the response of the gel energy storage modulus (G′) and loss modulus (G″), the test of a 0.1~100 Hz frequency range, and 0.2 Pa shear stress.

(3) Dehydration rate test. Temperature has a certain effect on the rheological behavior of the gel system. The CO_2_ response gel system was selected and placed in an oven at temperatures of 50 °C, 60 °C, and 70 °C, respectively. The dehydration mass was recorded at intervals for a total of 30 days and the dehydration rate was calculated.

### 4.5. Gas Channeling Plugging Capacity

A sandstone core 10 cm long and 2.5 cm in diameter was used with a permeability of 50 × 10^−3^ μm^2^. The sealing performance of the CO_2_ response gel plugging system and improvements in the effect of recovery were studied [43]. First, we determined the core dry weight, vacuum-extracted the formation water, determined the saturation to simulate, and calculated the core pore volume. A saturated oil experiment was then performed to record the saturated volume of oil. A CO_2_ displacement experiment was carried out at 70 °C, at a rate of 0.5 mL/min for CO_2_ flooding, with gas channeling out until exports. Then, we examined the response at a speed of 0.2 mL/min 0.5 PV of the gel system and waited for the gel formation. Finally, we released the pressure of the injection side, continued to use the 0.5 mL/min rate of CO_2_ gas displacement, recorded the change in the pressure, and calculated the total recovery factor and plugging rate [44].

## Figures and Tables

**Table 1:** Design and results of response surface experiments.

The No.	A	B	C	Y Intensity Score/(Points)
1	−1	−1	0	6.5
2	1	−1	0	6.6
3	−1	1	0	6.3
4	1	1	0	6.8
5	−1	0	−1	7.2
6	1	0	−1	7.4
7	−1	0	1	7.7
8	1	0	1	8
9	0	−1	1	8.5
10	0	1	−1	7
11	0	−1	1	7.5
12	0	1	1	8.9
13	0	0	0	9.6
14	0	0	0	9.3
15	0	0	0	9.4
16	0	0	0	9.2
17	0	0	0	9.5

**Table 2:** Regression model analysis of variance.

Source of Variation	Sum of Squares	Degree of Freedom	Mean Square	*F* Number	*p* Number	Significance
Model	21.71	9	2.41	157.08	<0.0001	**
A	0.1513	1	0.1513	9.85	0.0164	*
B	0.0012	1	0.0012	0.0814	0.7837	
C	0.5000	1	0.5000	32.56	0.0007	**
AB	0.0400	1	0.0400	2.60	0.1506	
AC	0.0025	1	0.0025	0.1628	0.6986	
BC	2.10	1	2.10	136.91	<0.0001	**
A^2^	11.12	1	11.12	723.99	<0.0001	**
B^2^	6.32	1	6.32	411.43	<0.0001	**
C^2^	0.1684	1	0.1684	10.97	0.0129	*
Residual	0.1075	7	0.0154			
Lack of Fit	0.0075	3	0.0025	0.1000	0.9559	
Pure Error	0.1000	4	0.0250			
Cor Total	21.82	16				
R^2^	0.9951					
R^2^_Adj_	0.9887					

Note: * indicates significant (*p* < 0.05), ** indicates extremely significant (*p* < 0.01).

**Table 3:** Experimental factors and levels of response surface.

Level	Factor		
	A Carrageenan Addition (wt%)	B Branched Polyvinylimide Addition (wt%)	C Ethylenediamine Addition (wt%)
−1	0.4	2.0	6.0
0	0.5	2.5	6.5
1	0.6	3.0	7.0

## Data Availability

The data presented in this study are openly available in the article.
